# QS-Net: Reconstructing Phylogenetic Networks Based on Quartet and Sextet

**DOI:** 10.3389/fgene.2019.00607

**Published:** 2019-07-24

**Authors:** Ming Tan, Haixia Long, Bo Liao, Zhi Cao, Dawei Yuan, Geng Tian, Jujuan Zhuang, Jialiang Yang

**Affiliations:** ^1^College of Computer Science and Electronic Engineering, Hunan University, Changsha, China; ^2^School of Information Science and Technology , Hainan Normal University, Haikou, China; ^3^Geneis (Beijing) Co. Ltd., Beijing, China; ^4^Department of Mathematics, Dalian Martine University, Dalian, China; ^5^Icahn Institute for Genomics and Multiscale Biology, Icahn School of Medicine at Mount Sinai, New York, NY, United States

**Keywords:** phylogenetic network, reticulate evolution, sextet, bacterial taxonomy, influenza reassortment

## Abstract

Phylogenetic networks are used to estimate evolutionary relationships among biological entities or taxa involving reticulate events such as horizontal gene transfer, hybridization, recombination, and reassortment. In the past decade, many phylogenetic tree and network reconstruction methods have been proposed. Despite that they are highly accurate in reconstructing simple to moderate complex reticulate events, the performance decreases when several reticulate events are present simultaneously. In this paper, we proposed QS-Net, a phylogenetic network reconstruction method taking advantage of information on the relationship among six taxa. To evaluate the performance of QS-Net, we conducted experiments on three artificial sequence data simulated from an evolutionary tree, an evolutionary network involving three reticulate events, and a complex evolutionary network involving five reticulate events. Comparison with popular phylogenetic methods including Neighbor-Joining, Split-Decomposition, Neighbor-Net, and Quartet-Net suggests that QS-Net is comparable with other methods in reconstructing tree-like evolutionary histories, while it outperforms them in reconstructing reticulate events. In addition, we also applied QS-Net in real data including a bacterial taxonomy data consisting of 36 bacterial species and the whole genome sequences of 22 H7N9 influenza A viruses. The results indicate that QS-Net is capable of inferring commonly believed bacterial taxonomy and influenza evolution as well as identifying novel reticulate events. The software QS-Net is publically available at https://github.com/Tmyiri/QS-Net.

## Introduction

Phylogenetic tree is usually utilized to show the evolutionary history of a set of biological entities or taxa. However, the tree-like topology cannot represent reticulate evolutionary events, such as horizontal gene transfer (HGT), hybridization, recombination, or reassortment, which have been shown to be critical in genotypic diversity, related phenotypes, estimations of evolutionary history, and virus emergence and immune evasion (Fenderson and Bruce, 2008; Vijaykrishna et al., 2015; Bastide et al., 2018). For example, HGT, also known as lateral gene transfer (LGT), promotes the diversification of microorganisms on the evolutionary time scale. This mechanism can change the types and characteristics of bacteria and plays a major role in the genetic diversity of bacteria (Ochman et al., 2000). In the long run, it may be the dominant force affecting genes in most prokaryotes. Recombination is a major source of genotypic diversity and a core force for the formation of genome and related phenotypes (Leducq et al., 2017). Reassortment is responsible for most antigenic shifts of influenza virus (Nelson et al., 2008). Hybridization has been shown to be the main evolutionary mechanism for plants and some animals (Rieseberg et al., 2000; Yu et al., 2011).

A phylogenetic network can serve as an alternative to phylogenetic tree. When the evolutionary history of a sequence set contains reticulate events (Huson et al., 2010), generally speaking, phylogenetic networks can be divided into explicit and implicit networks. The implicit phylogenetic networks, such as split network, are often adopted to illustrate incompatible data and capture conflicting signals in a data set. With the increasing sequencing data, phylogenetic networks have become more and more important in molecular evolution.

Over the past decades, many methods have been proposed for reconstructing phylogenetic trees or networks. The most common type of method reconstructs a network directly from the original character data, usually through a parsimony or maximum-likelihood criterion. Methods in this category include Spectronet (Huber et al., 2002), maximum pseudo-likelihood estimation (Yu and Nakhleh, 2015), HGT maximum parsimony (Park et al., 2010), PhyloNetwork (Solís-Lemus et al., 2017), inferring phylogenetic networks using PhyloNet (Wen et al., 2018), and SNaQ (Claudia and Cécile, 2016). However, these methods are inefficient computationally and tend to overestimate the actual number of reticulate events in the evolutionary history (Huelsenbeck, 1995; Park et al., 2010). The second widely used method is the distance-based method, which first builds a genetic distance matrix for a taxa set and then reconstructs the phylogenetic network from the distance matrix. Methods in this category include Neighbor-Net (Bryant and Moulton, 2004), Split-Decomposition (Bandelt and Dress, 1992), FastME (Lefort et al., 2015), ASTRID (Vachaspati and Warnow, 2015), tree-average distances method (Willson, 2013), and large-scale Neighbor-Joining with NINJA (Wheeler, 2009). The distance-based methods are very fast compared with character-based methods, but they have a disadvantage in terms of reconstruction accuracy. The third kind of methods reconstructs phylogenetic networks from weighted triplets and quartets because they can retain more information than distances. Methods in this category include local maximum likelihood using triplets (Ranwez and Gascuel, 2002), Quartet-Net (Yang et al., 2013), tree with strong combinatorial evidence (Berry and Gascuel, 2000), QNet (Grünewald et al., 2007), SuperQ (Grunewald et al., 2013), DistiQue (Sayyari and Mirarab, 2016), level 1 network from a dense quartet (Keijsper and Pendavingh, 2014), and weighted QMC (Avni et al., 2015). In addition, there are other methods using statistical models such as stochastic local search method (Tria et al., 2010), clusters (Van Iersel et al., 2010), Bayesian inference (Zhang et al., 2017), statistical model (Pickrell and Pritchard, 2012), and Monte Carlo method (Eslahchi et al., 2010).

Quartet-Net (Yang et al., 2013) is a method for reconstructing phylogenetic networks from a set of weighted triplets and quartets, which uses parsimony information sites to calculate triplet and quartet weights directly from multiple sequence alignment (MSA). Based on the calculated triplet and quartet weights, Quartet-Net then performs a split expanding process to obtain all full splits and their weights, which will transform to an evolutionary tree or network. The method is a generalization of Split-Decomposition (Bandelt and Dress, 1992). In this paper, we further generalize Quartet-Net and propose a novel method called QS-Net to reconstruct evolutionary networks based on weighted quartets and sextets. The analysis of artificial and real data sets shows that this method can reconstruct a more accurate phylogeny when the sequence data are generated from complicated evolutionary scenarios involving many reticulate events and identifies novel reticulate evolution and reassortment events.

## Materials and Methods

### Background: Split and Split Weight

**Figure 1:**
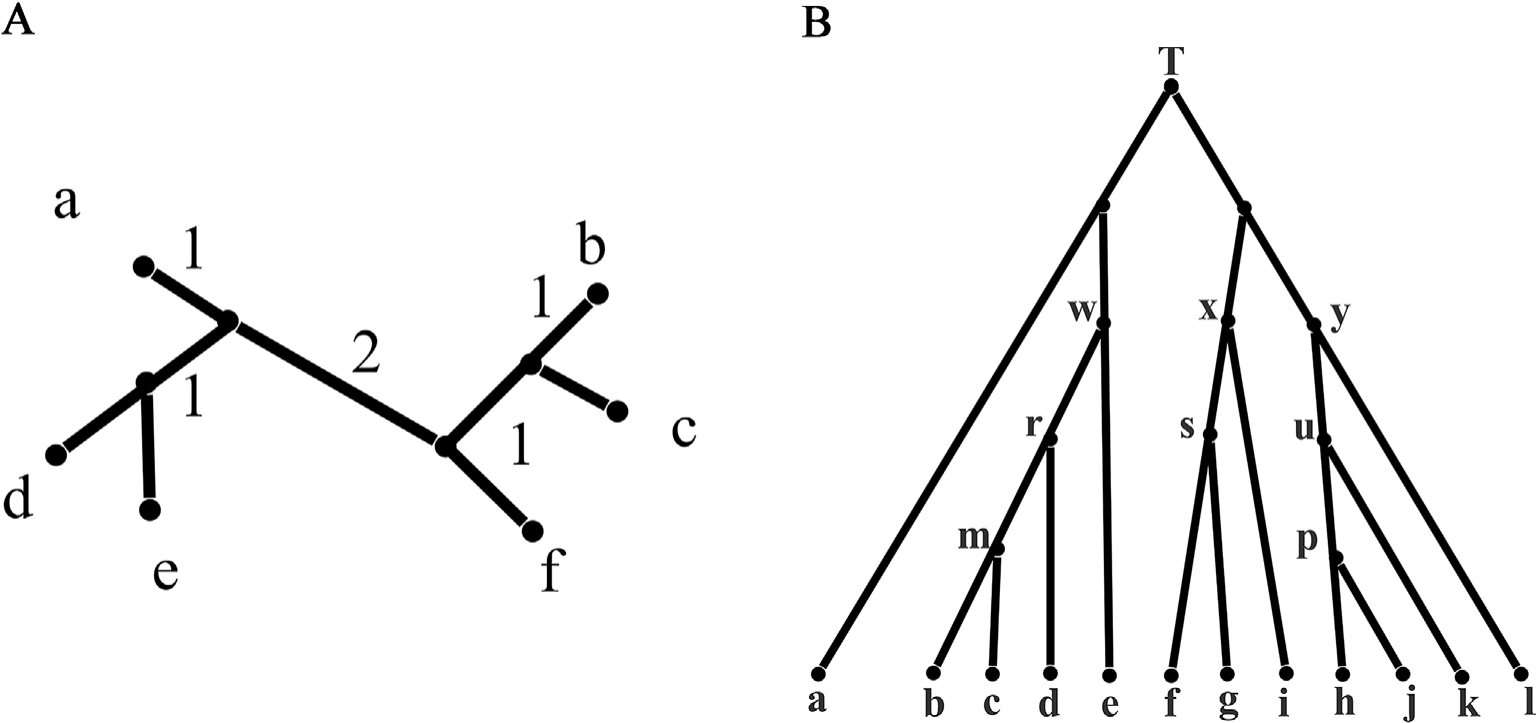
Phylogenetic tree: a phylogenetic tree for illustration and a phylogenetic tree with 12 leaves. **(A)** A phylogenetic tree for illustration with the branch length indicating evolutionary distance. **(B)** A phylogenetic tree with 12 leaves used to generate the first simulation data.

For a taxa set S = {S_1_, S_2_,…,S_n_} of size n, a split consisting of two disjoint non-empty subsets of S is denoted by A | B that is, A and B. If A and B contain all the taxa in S, then A | B is called a full split; otherwise, it is called a partial split. In a phylogenetic tree, each edge is a full split that divides the tree into two parts, while in a phylogenetic network, a group of parallel edges with equal length represents a full split. If |A| = 1 or|B| = 1, the split A|B is called a trivial split. For example, the phylogenetic tree in **Figure 1A** contains five trivial full splits, such as a|bcdef, and three non-trivial full splits de|abcf, bc|adef, and ade|bcf. In general, a split A|B with |A| = m and |B| = n is called an m|n split. In addition, W(A|B) represents the evolutionary distance between taxa groups A and B. If A or B contains more than two taxa, then W(A|B) calculates the distance between the common ancestor of A and B. For example, W(a|de) = 2, W(d|ae) = 1 in **Figure 1A**, W(a|d) represents the evolutionary distance between taxa a and d, and therefore, through **Figure 1A** and these definitions, we can get this equation W(a|d) = W(a|de) + W(ae|d).

For an MSA, a simple parsimony-based method is used to estimate the weights of quartets and sextets. For example, if the character in a site is the same for taxa a, b, and c and for taxa d, e, and f, but different for a and d, then the site is defined to support the split abc | def. For any sextet abc | def, its weight W(abc|def) is defined to be the proportion of total number of sites supporting it in the MSA. The weight of a quartet say ab|cd is calculated in a similar way. After all the quartet and sextet weights are obtained, an ever-expanding process is performed based on these weights to all full splits and their weights. As shown in previous literatures (Bandelt and Dress, 1992; Yang et al., 2013), reconstructing a phylogenetic tree or network is equivalent to calculating all the full splits and their weights. Thus, we have obtained the reconstructed tree or network by this process, which could be shown by a software SplitsTree4 (Huson and Bryant, 2006).

### Ever-Expanding Process Based on Quartet and Sextet Weights

As represented by equation W(a|d) = W(a|de) + W(ae|d), there is such an equation W(abc|def) = W(abc|defg) + W(abcg|def), which can be seen as adding a new taxon g to either side of a split abc|def. If W(abc|def) = 0, then W(abc|defg) = 0 and W(abcg|def) = 0. If taxa group A_1_ ⊆ A and B_1_ ⊆ B, or A_1_ ⊆ B and B_1_ ⊆ A, we call the split A|B displays A_1_|B_1_. It is proven in Bandelt and Dress (1992) that W(A|B) ≤ W(A_1_|B_1_). Therefore, a split with zero weight cannot be further expanded to larger splits with positive weights.

For a taxa set S with size n, there are $10{(}_{6}^{\text{n}})$ sextets. We first calculate the weights of all quartets and sextets from the MSA, and then we expand them to get all full split weights using an ever-expanding process. Suppose there is a septet of abc|defg type, we have W(abc|defg) = W(abc|def) − W(abcg|def), and there is a similar equation for W(abcg|def), so the weight of W(abc|defg) can be obtained by similar continuous calculations, as follows.

$$\{\begin{array}{l} \text{W(abc|defg)}=\text{W(abc|def)}-\text{W(abcdg|def)}\\ \text{W(abcg|def)}=\text{W(abg|def)}-\text{W(abg|cdef)}\\ \text{W(abg|cdef)}=\text{W(abg|cde)}-\text{W(abfg|cde)}\\ \text{W(abfg|cde)}=\text{W(afg|cde)}-\text{W(afg|bcde)}\\ \text{W(afg|bcde)}=\text{W(afg|bcd)}-\text{W(aefg|bcd)}\\ \text{W(aefg|bcd)}=\text{W(efg|bcd)}-\text{W(efg|abcd)}\\ \text{W(efg|abcd)}=\text{W(efg|abc)}-\text{W(defg|abc)} \end{array}$$

Combining the above equations, we have

(1) $$\begin{array}{l} \text{W}(abc|defg)=\frac{1}{2}\{W(abc|def)-W(abg|def)+\\ W(abg|\text{cde})-\text{W}(afg|cde)+\text{W}(afg|bcd)-\\ W(efg|bcd)+W(efg|abc)\} \end{array}$$

For |B| ≥ 4, taking minimum over all possible cases, we have

(2) $$\begin{array}{l} \text{W}(abc\text{|B})=\max\{\frac{1}{2}\underset{\text{defg }\in \text{ B}}{\min}\{W(abc\text{|}def)-W(abg\text{|}def)+\\ W(abg\text{|cde})-\text{W}(afg\text{|}cde)+\\ \text{W}(afg\text{|bcd})-\text{W(efg|bcd)}+\text{W(efg|abc)\},0}\} \end{array}$$

When |A|=4 and |B|=4, the weight of the 4|4 split

(3) $$\begin{array}{l} \text{W}(\text{A|B})=\min\{\underset{\text{a }\in \text{ A}}{\min}\{W(\text{A}-a\text{|B})-W(\text{A}-a\text{|B }+a)\},\\ \;\underset{\text{a }\in \text{ B}}{\min}\{W(\text{A|B}-\text{e)}-\text{W(A+e|B}-\text{e)}\} \end{array}$$

where A−A′ for two sets A and A′ denotes set difference (subtraction).

For example A={a, b, c, d}, B={e, f, g, h}, there are eight equations for W(abcd|efgh),

$$\text{W(abcd|efgh)}=\{\begin{array}{l} \text{W(abc|efgh)}-\text{W(abc|defgh)}\\ \text{W(abd|efgh)}-\text{W(abd|cefgh)}\\ \text{W(acd|efgh)}-\text{W(acd|befgh)}\\ \text{W(bcd|efgh)}-\text{W(bcd|aefgh)}\\ \text{W(abcd|efg)}-\text{W(abcdh|efg)}\\ \text{W(abcd|efh)}-\text{W(abcdg|efh)}\\ \text{W(abcd|egh)}-\text{W(abcdf|egh)}\\ \text{W(abcd|fgh)}-\text{W(abcd|fgh)} \end{array}$$

For any split A|B with |A| ≥ 4 and |B| ≥ 4, we traverse the elements in A and B and take out four taxa for each calculation. Suppose a, b, c, d ∈ A and e, f, g, h ∈ B, and we have

(4) $$\text{W(A|B)}=\underset{\text{abcd }\in \text{ A; efgh }\in \text{ B}}{\min}\{W(abcd|efgh)\}$$

For any 2|n split of ab|B type with c, d, e ∈ B, we calculate their weight by formula (5) referred in Quartet-Net (Yang et al., 2013),

(5) $$\begin{array}{c} \text{W(ab|B)}=\max\{\frac{1}{2}\underset{\text{cde }\in \text{ B}}{\min}\{W(ab\text{|cd})-\text{W(ae|cd)}+\\ \text{W(ae|bc)}-\text{W(bc|de)+W(ab|de)}\},0\} \end{array}$$

Finally, for any trivial split of a|S − a type with b, c ∈ S−a in a taxa set S, we calculate the weight as follows (see also Yang et al., 2013):

(6) $$\text{W(a|S}-\text{a)}=\underset{\text{bc }\in \text{ S}-\text{a}}{\min}\left\{\text{W(a|bc)}-\sum_{\text{a }\in \text{ A; bc }\in \text{ B}}\text{A|B}\right\}$$

Formulas (1) – (6) are used to calculate all full splits by decomposing sextet weights iteratively.

### The QS-Net Method

QS-Net takes an MSA as input. Suppose that there are n taxa in the taxa set S, which are arranged in the order of 1, 2, 3, …, n. In the initialization step, all triplet, quartet, and sextet weights are calculated directly from the MSAs. We calculate the weights of full splits in the following ways.

- Full split of type A|S − A with |A| ≥ 3 and |S−A| ≥ 3: for the first six taxa—1, 2, 3, 4, 5, and 6—there are 10 sextets. We store these sextets together with their weights in a set X_1_. QS-Net then iteratively adds i=7, 8…, n to the left and right parts of the splits stored in X_1_ and use equations (2)–(4) to calculate the weights of newly generated splits. Noticing that the only splits that cannot be generated in this way are of type i j k|S_1_ − {i, j, k} with j = i − 1, i – 2, …, 2 and k = j – 1, j − 2, …, 1, we calculate their weights using equation (2) and add them to X_1_. At the end of each iteration, the splits with a weight of zero are removed because they cannot be further expanded to have a positive weight. After the last iteration, all full splits of type |A| ≥ 3 and |S − A| ≥ 3 have been calculated.
- 2|n – 2 full splits: These splits can be calculated using equation (5). In practice, we use Quartet-Net to calculate their split weights.
- Trivial (1|n − 1) full splits: These splits can be calculated by equation (6).

By the above procedures, we calculate the weights of all full splits. Similar to Yang et al. (2013), it is usually advisable to filter the non-trivial full splits with very low split weights, which tend to be false positives. In practice, we remove splits with weight less than c% of the average weight, where c is a user-defined threshold setting to be 1 in this study. The output file containing all non-zero full splits and their weights is stored in.NEXU file format, which can be visualized using SplitsTree4 (Huson and Bryant, 2006). The time complexity of QS-Net is O(n^10^).

## Results And Discussions

To demonstrate QS-Net, we analyzed three artificial data sets and two real data sets. The artificial data sets were generated from a simple tree phylogeny, a phylogenetic scenario with three reticulate events, and a more complicated phylogenetic scenario with five reticulate events. The purpose is to show that the QS-Net method can accurately reconstruct all kinds of evolutionary histories from simple to complicated ones. The real data include a bacterial taxonomy data consisting of 36 bacterial species and the whole genome sequences of 22 H7N9 influenza A viruses downloaded from NCBI influenza database.

The software Dawg (Cartwright, 2005) with model GTR + Gamma + I was used to generate three artificial data sets. The substitution rate is 0.01; the sequence length of the tree is 10,000 bp; the sequence length of the network containing three evolutionary events is 80,000 bp, while the sequence length of the network containing five evolutionary events is 320,000 bp because they are a concatenation of eight and 32 feasible trees. To avoid randomness, we performed 100 Dawg runs on each of the three artificial data sets and applied the 100 MSAs of each data set to QS-Net together with other four popular methods: Quartet-Net (Yang et al., 2013), Neighbor-Net (Bryant and Moulton, 2004), Split-Decomposition (Bandelt and Dress, 1992), and Neighbor-Joining (Saitou and Nei, 1987).

### Analysis on the Tree Data

The tree data were generated from **Figure 1B** with 12 leaves. For brevity, we only listed reconstructed taxa set in the left or right block containing fewer number of taxa (**Supplementary Material: Table S1**). For example, split bd|acefghijkl was listed as bd. We then normalized each split by the weight of a split successfully constructed by all methods. All trivial full splits were not listed because they can be successfully reconstructed by all five methods. As shown in **Table 1**, all five methods can successfully reconstruct all full splits in the 100 runs of the tree data; the accuracy is equal to the experimental bootstrap value divided by the real bootstrap value. The true-positive split result represents all splits in the real phylogenetic history of the simulated data sets. We listed the number of true-positive splits obtained by the five methods on all simulated data sets in **Table 2**. If a method can reconstruct the true-positive split once in 100 runs, we determined that the true-positive split can be obtained by this method. In addition to true-positive results, other split results reconstructed by the method are false-positive splits, which typically have very few weight values. Except for Neighbor-Joining, the other four methods reconstructed some false-positive splits (here we only list false-positive splits with a bootstrap value ≥10). For example, Quartet-Net and QS-Net reconstruct two additional split al and ae with bootstrap values of 10 and 26, respectively (see **Table 3**). This is because QS-Net and Quartet-Net methods use the same calculation formula for split of 2|n type. Neighbor-Net identifies 35 false-positive splits with bootstrap value ranging from 10 to 40. These false-positive splits may be caused by some random mutations in the tree data set.

**Table 1:** Comparison of accuracy (the total bootstrap value obtained from the experimental results is divided by the bootstrap BV value) between QS-Net and four other methods.

Data set	QS-Net	Quartet-Net	Neighbor-Net	Split-Decomposition	Neighbor-Joining
Tree	100%	100%	100%	100%	100%
Network (3)	100%	100%	70.16%	67.24%	36%
Network (5)	100%	94.74%	58.89%	46.76%	23.68%

Network (3) is the phylogenetic network with three reticulate events, while Network (5) is the phylogenetic network with five reticulate events.

**Table 2:** The number of true-positive results can be obtained by five methods.

Data set	True	QS-Net	Quartet-Net	Neighbor-Net	Split-Decomposition	Neighbor-Joining
Tree	9	9	9	9	9	9
Network (3)	25	25	25	21	23	9
Network (5)	38	38	36	30	22	11

The “True” column represents the real number of true-positive splits of the simulated data.

**Table 3:** The number of false-positive results obtained by five methods.

Data set	QS-Net	Quartet-Net	Neighbor-Net	Split-Decomposition	Neighbor-Joining
Tree	2	2	35	4	0
Network (3)	4	4	16	1	0
Network (5)	4	4	4	1	0

### Analysis on the Network Data with Three Reticulate Events

**Figure 2:**
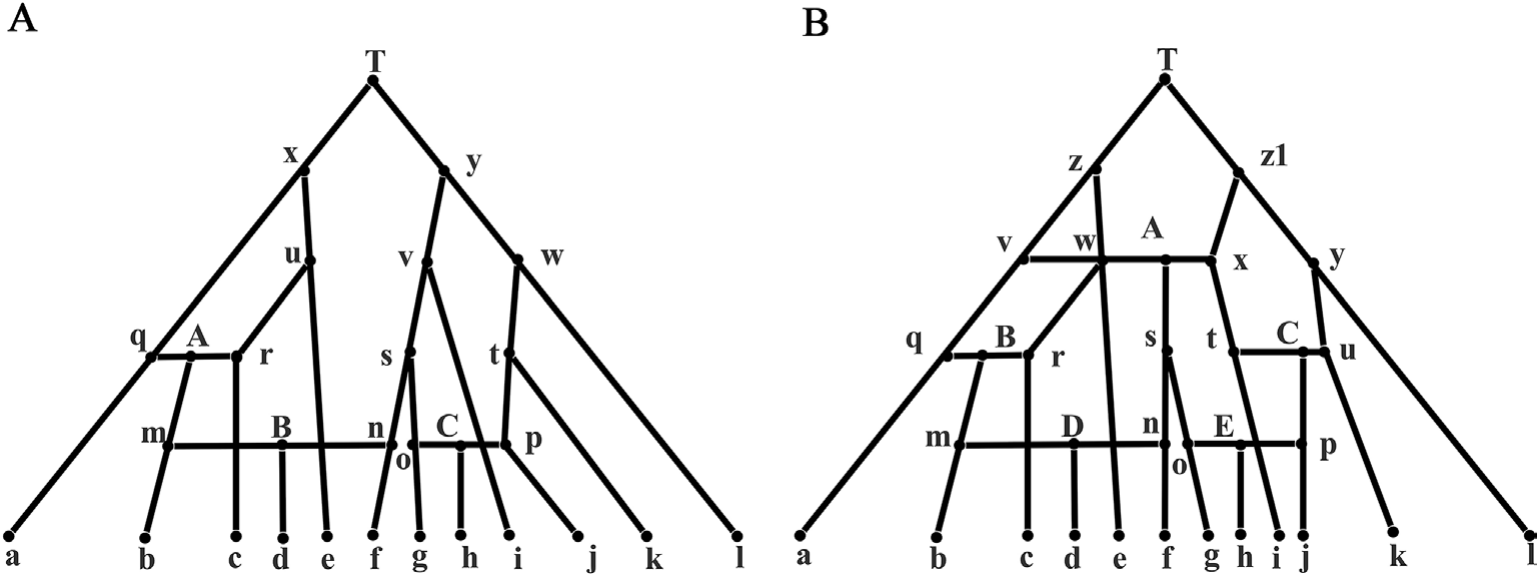
Phylogenetic network with 3/5 reticulate events. **(A)** A phylogenetic network with three reticulate events A, B, and C. **(B)** A phylogenetic network with five reticulate events A, B, C, D, and E.

The network data were generated from **Figure 2A** containing three reticulate events A, B, and C, which can be decomposed into eight feasible underlying trees. A feasible tree can be obtained by cutting off one branch respectively at A, B, and C. For example, we can get an underlying tree by cutting off the three edges qA, mB, and oC in the three reticulate events. The sequence data of a taxon m were generated by concatenating partial sequence data from q and partial sequence data from r. All true splits and splits reconstructed by the five methods are listed in **Supplementary Material: Table S2**. The weight of the true split is the sum of the split weights in eight feasible trees. Similarly, we normalized each split with the weight of split ab and multiplied it by 4. As can be seen from the **Table 1**, QS-Net and Quartet-Net accurately reconstructed all true splits in all 100 runs, while Neighbor-Net, Split-Decomposition, and Neighbor-Joining failed to reconstruct a large number of true splits. For example, Neighbor-Net failed to reconstruct split gh, fgi, and fgh in more than 90 runs, and Split-Decomposition was unable to reconstruct split bce and bcde in all 100 runs (**Supplementary Material: Table S2**). Neighbor-Joining obtained even worse result with 16 true splits missing, which is reasonable because Neighbor-Joining only reconstructs trees and retains the strongest compatible splits.

### Analysis on the Network Data with Five Reticulate Events

**Supplementary Material Table S3** lists all true splits and splits reconstructed from the five methods from the network data. The data set was generated from **Figure 2B** with a complicated phylogenetic scenario containing five reticulate events. Similarly, the weight of the true split is the sum of the weights of the splits in 32 feasible trees. We normalized each split with the weight of split ce. As can be seen from the **Table 1**, only QS-Net method obtains 100% accuracy in all 100 runs, while the other four methods fail to reconstruct some splits in most runs. For example, Quartet-Net failed in reconstructing split fgi and afg in all 100 runs. In addition to the two splits, Neighbor-Net also cannot reconstruct split hj, bcd, and bcde in more than 90 runs (**Supplementary Material: Table S3**), which happens because Neighbor-Net reduces splits to make the split system planar. Split-Decomposition and Neighbor-Joining still performed poorly. In addition, all methods except for Neighbor-Joining reconstructed some false-positive splits.

### Analysis on the Bacterial Data

**Figure 3:**
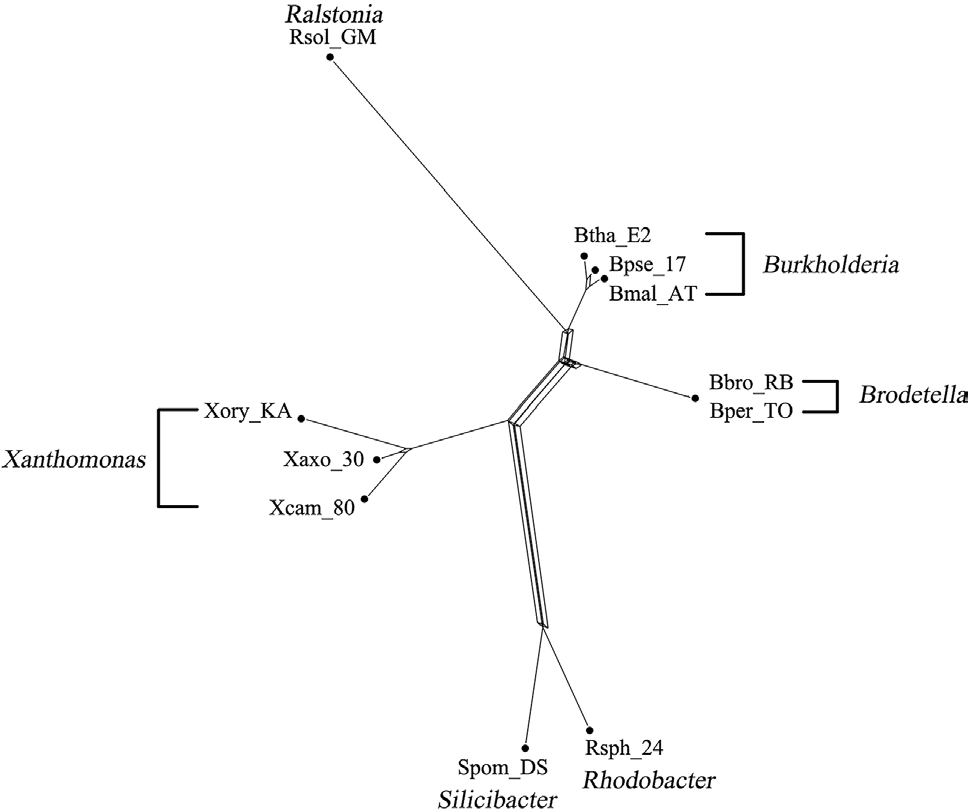
The reconstructed QS-Net network of 11 GC-rich bacteria.

**Figure 4:**
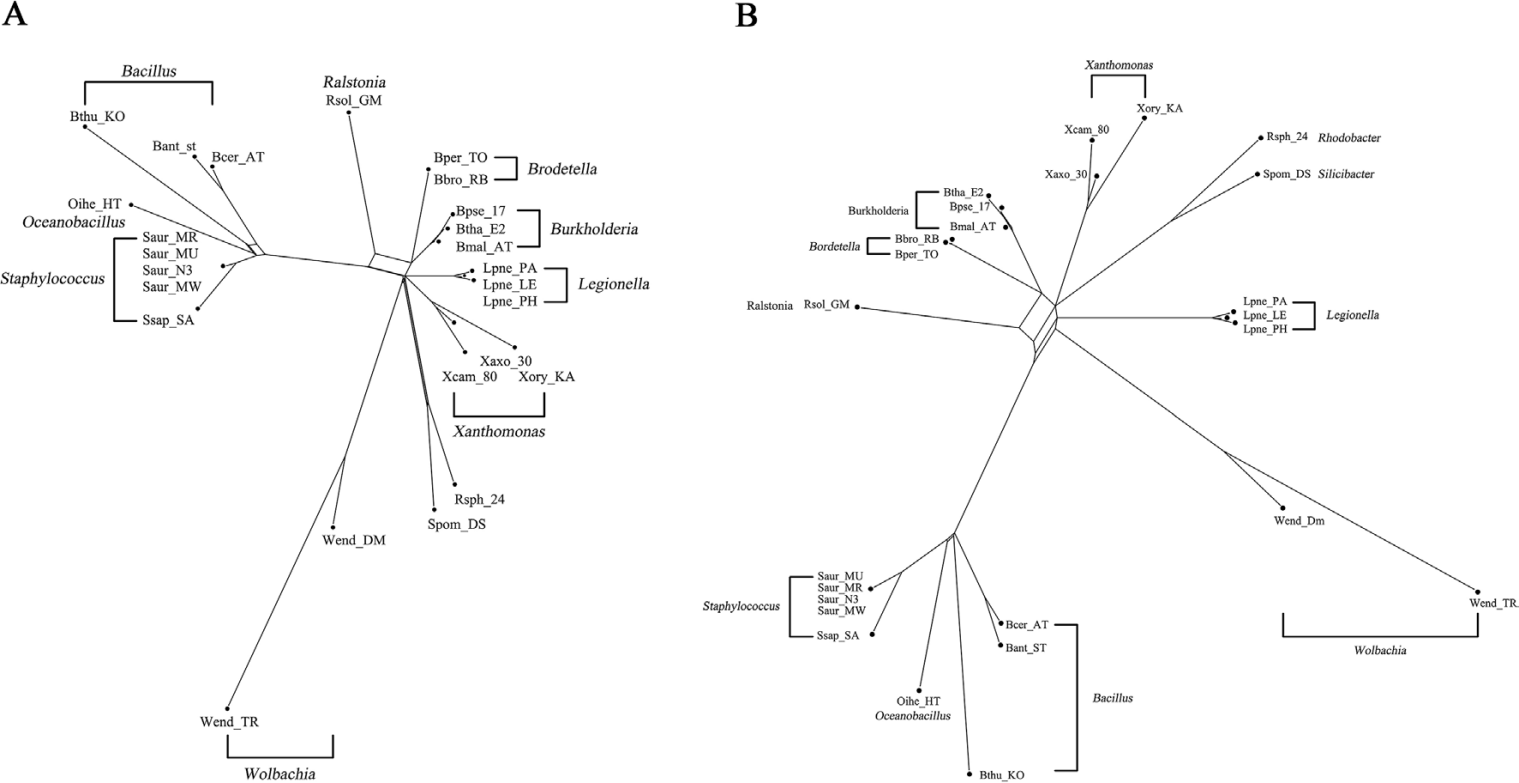
The reconstructed network on 25 GC-poor or GC-rich bacteria. **(A)** The reconstructed QS-Net network of 25 GC-poor or GC-rich bacteria. **(B)** The reconstructed Quartet-Net network of the 25 bacteria.

The bacterial data set was used in Takahashi and Kryukov (2009) for the analysis of phylogenetic relationships among bacterial species. This data set consists of 36 bacterial genomes containing concatenated sequence of seven genes (16S rRNA, 23S rRNA, gyrB, pyrH, recA, rpoA, and rpoD). The 36 species were divided into three different groups based on different GC content (32–38%, 50–53%, and 64–69%), containing 14, 11, and 11 species, respectively. We took the GC-rich data consisting of 11 bacterial species and a data of 25 species containing both GC-poor and GC-rich bacteria. The MSAs of both data were generated by ClustalW (Larkin et al., 2007) and further fed into to QS-Net, Quartet-Net (Yang et al., 2013), Neighbor-Net (Bryant and Moulton, 2004), Split-Decomposition (Bandelt and Dress, 1992), and Neighbor-Joining (Saitou and Nei, 1987). We ran the program on an MSI laptop with 2.8-GHz processor and 8-GB memory. A comparison of runtime between QS-Net and Quartet-Net on all data sets is shown in **Table 4**; the time statistics for three artificial data sets are the average of all 100 runtimes. The Neighbor-Joining method has the least runtime, and all other three methods can produce results in less than 2 s on all data sets. The reconstructed results were then viewed by SplitsTree4 (Huson and Bryant, 2006). Only three split networks reconstructed by QS-Net and Quartet-Net method on bacterial data set are shown in **Figures 3** and **4**.

**Table 4:** A comparison of runtime between QS-Net and Quartet-Net on all data sets.

Method	Tree	Network (3)	Network (5)	GC rich	GC poor and rich	Influenza
QS-Net	1.25 s	6.02 s	24.39 s	0.92 s	9.49 min	3.22 min
Quartet-Net	0.20 s	1.05 s	4.05 s	0.19 s	10.17 s	4.54 s

**Figure 3** shows the phylogenetic network of 11 GC-rich bacterial sequence data set by using QS-Net, which is basically consistent with the experimental results in Takahashi and Kryukov (2009). The reconstructed networks of 25 GC-poor or GC-rich (32–38% and 64–69%) sequence data set reconstructed by QS-Net and Quartet-Net are shown in **Figures 4A, B**, respectively. As can be seen from the figures, the differences between QS-Net and Quartet-Net are quite obvious. There are two distinct parallelograms that represent the reticulate evolution event in the reconstructed network in **Figure 4A** but not in **Figure 4B**, which might be neglected by Quartet-Net due to its inability to identify complicated reticulate events. The numbers of full splits reconstructed by the five methods on bacterial data set and the influenza data set are also listed in **Table 5**. QS-Net constructs a moderate total number of splits among all comparison methods, probably because the full resolution of taxa is not achieved. In the GC-rich data set, Neighbor-Net constructs three more splits than does QS-Net, while in the GC-poor and GC-rich data set, Neighbor-Net constructs 29 more splits than does QS-Net. In addition, by comparing **Figures 3** and **4A**, it can be found that GC content may have an important influence on the evolutionary history of bacteria.

**Table 5:** The number of full splits reconstructed by five methods on bacterial data set and the influenza data set.

Data set	QS-Net	Quartet-Net	Neighbor-Net	Split-Decomposition	Neighbor-Joining
GC rich	26	22	29	23	19
GC poor and rich	48	45	77	48	47
Influenza	47	45	68	36	41

### Analysis on the Influenza Data

**Figure 5:**
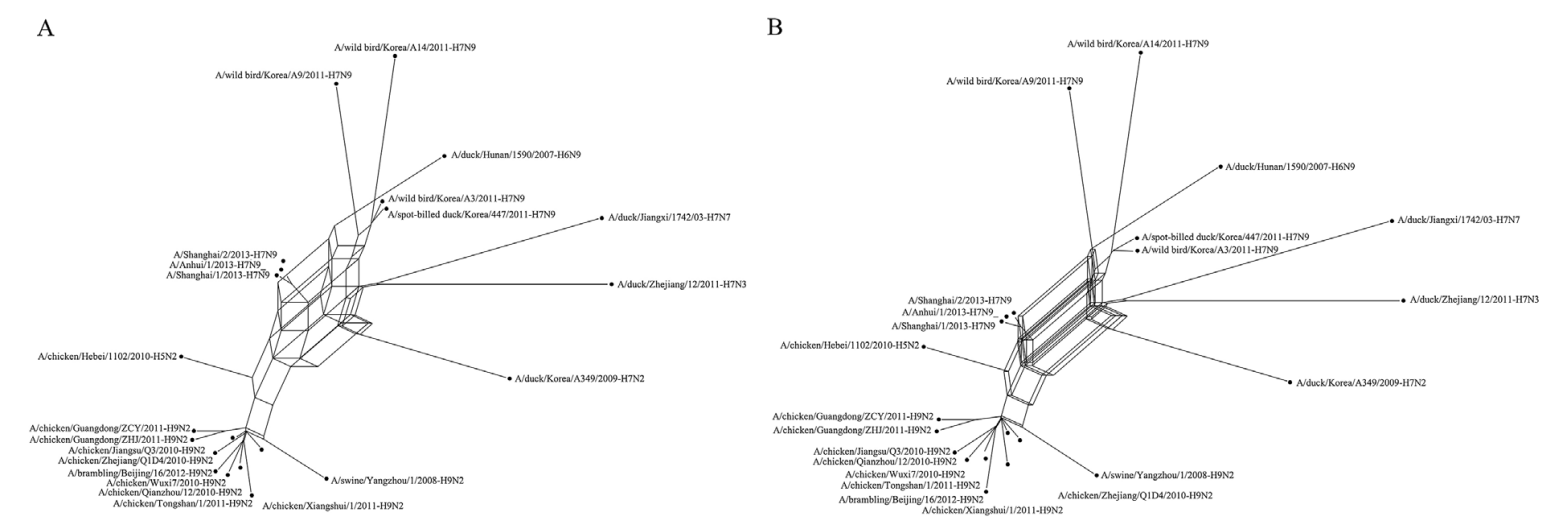
The reconstructed network on influenza data. **(A)** The reconstructed Quartet-Net network related to H7N9 influenza A viruses. **(B)** The reconstructed QS-Net network related to H7N9 influenza A viruses.

The data set consisted of the full genome sequence of 22 H7N9 influenza A viruses aligned by ClustalW (Larkin et al., 2007). These viruses have major relations with the H7N9 virus (Gao et al., 2013) that appeared in China in 2013, which caused human mortality. We estimated the phylogenetic relationships of these 22 influenza A viruses using Quartet-Net and QS-Net. The results are shown in **Figures 5A, B**, respectively. **Table 5** lists the numbers of full splits reconstructed by the five methods on bacterial data set and the influenza data set. General split networks do not actually represent explicit evolutionary events, which makes the interpretation and comparison of reconstruction methods on real data set difficult. So we list the number of splits built by various methods. As can be seen in **Table 4**, QS-Net reconstructs 47 full splits, while Quartet-Net reconstructs 45 full splits.

The three viruses that caused human death (A/Shanghai/1/2013, A/Shanghai/2/2013, and A/Anhui/1/2013) were combined. The phylogenetic network indicates that these H7N9 viruses may be derived from the reassortment from influenza subtypes, including avian-origin H7N9 viruses, H9N2 viruses, and H7N3 viruses. In **Figure 5B** (constructed by QS-Net), the internal region surrounded by H7N9, H7N7, and H7N3 is more complex than **Figure 5A** (constructed by Quartet-Net), which indicates that the true evolutionary history of H7N9 influenza A viruses is very complex. Of course, the real evolutionary history is unknown, but at least the results constructed by QS-Net are consistent with a few previous findings.

## Conclusions

QS-Net is a method generalizing Quartet-Net. Both simulation studies and real data analyses show that QS-Net has the potential to reconstruct more accurate phylogenetic relationships than its competitors like Quartet-Net and Neighbor-Net. However, the method runs slower than other algorithms, and the major computational difficulty lies in the calculation of 3|4 splits. Nevertheless, the difficulty will be partially resolved with the development of high-speed computers and parallel algorithms. Thus, we believe QS-Net will be useful in identifying more complex reticulate events that will be ignored by other network reconstruction algorithms.

## Author Contributions

JY and BL conceived the concept of the work and designed the experiments. MT, HL, ZC, and JZ performed literature search. MT, HL, DY, and GT collected and analyzed the data. MT and JY wrote the paper. All authors have approved the final manuscript.

## Funding

This work was supported by Hainan Provincial Innovation research team (No. 2019CXTD405), National Natural Science Foundation of China (No. 61762034), Hainan Provincial Natural Science Foundation of China (No.618MS057, No.617122) , Hainan Provincial major scientific and technological plans (No.ZDKJ2017012), Natural Science Foundation of Hunan, China (Nos. 2018JJ2461 and 2018JJ3568), New Century Excellent Talents in university (No. NCET-10-0365), National Nature Science Foundation of China (Nos 11171369, 61272395, 61370171, 61300128, 61472127, 61572178, 61672214, and 61702054).

## Conflict of Interest Statement

Authors DY and GT were employed by company Geneis (Beijing) Co. Ltd. The remaining authors declare no competing interests.
