# Supplementary material for: QS-Net: Reconstructing Phylogenetic Networks Based on Quartet and Sextet
Source: Front Genet. 2019 Jul 24;10:607. doi: 10.3389/fgene.2019.00607 (PMC6667645; doi:10.3389/fgene.2019.00607)
Supplement: Supplementary file 1 [file Table_1.docx]

Supplementary Material

QS-Net: Reconstructing Phylogenetic Networks based on Quartet and Sextet

Ming Tan^1#^, Haixia Long^2#^, Bo Liao^1,2*^, Zhi Cao^1,*^, Dawei Yuan^3^, Geng Tian^3^, Jujuan Zhuang^4^ and Jialiang Yang^2,5*^

^1^College of Computer Science and Electronic Engineering, Hunan University, Changsha, Hunan, 410082, P.R. China

^2^School of Mathematics and Statistics, Hainan Normal University, Haikou, 570100, P.R. China

^3^Geneis (Beijing) Co. Ltd., Beijing 100102, P.R. China

^4^Department of Mathematics, Dalian Maritime University, Dalian, Liaoning 116026, P. R. China

^5^Icahn Institute for Genomics and Multiscale Biology, Icahn School of Medicine at Mount Sinai, New York, NY 10029, USA

**^#^** The authors contributed equally to this study

*** Correspondence:**

Bo Liao [dragonbw@163.com](mailto:dragonbw@163.com)

Zhi Cao [66384436@qq.com](mailto:66384436@qq.com)

Jialiang Yang [jialiang.yang@mssm.edu](mailto:jialiang.yang@mssm.edu)

# Supplementary Tables

**
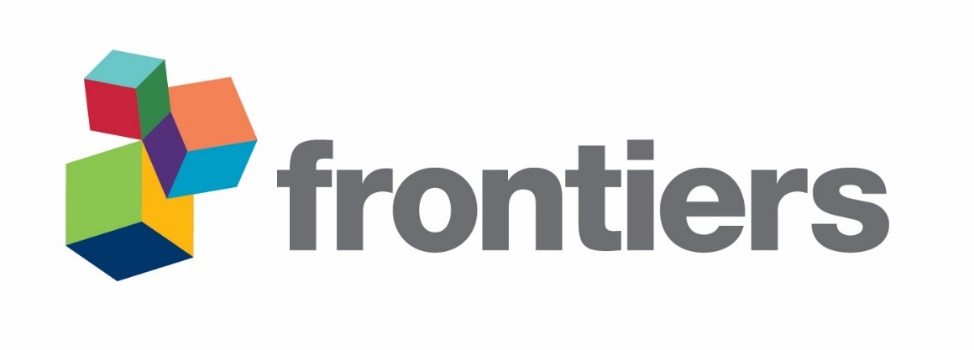
**

**Supplementary Table S1.** True splits and splits reconstructed from the tree data by five methods.

| **True** | | |  | **QS-Net** | | |  | **Quartet-Net** | | |  | **Neighbor-Net** | | |  | **Split-Decomposition** | | |  | **Neighbor-Joining** | | |
| --- | --- | --- | --- | --- | --- | --- | --- | --- | --- | --- | --- | --- | --- | --- | --- | --- | --- | --- | --- | --- | --- | --- |
| **Split** | **WV** |  | | **Split** | **WV** | **BV** |  | **Split** | **WV** | **BV** |  | **Split** | **WV** | **BV** |  | **Split** | **WV** | **BV** |  | **Split** | **WV** | **BV** |
| bc | 1 |  | | bc | 1 | 100 |  | bc | 1 | 100 |  | bc | 1 | 100 |  | bc | 1 | 100 |  | bc | 1 | 100 |
| fg | 1 |  | | fg | 0.99 | 100 |  | fg | 0.99 | 100 |  | fg | 0.97 | 100 |  | fg | 0.97 | 100 |  | fg | 1.02 | 100 |
| hj | 1 |  | | hj | 1.01 | 100 |  | hj | 1.01 | 100 |  | hj | 1.01 | 100 |  | hj | 1.01 | 100 |  | hj | 1.03 | 100 |
| hjk | 1 |  | | hjk | 0.94 | 100 |  | hjk | 0.98 | 100 |  | hjk | 0.98 | 100 |  | hjk | 0.97 | 100 |  | hjk | 0.99 | 100 |
| fgi | 1 |  | | fgi | 0.92 | 100 |  | fgi | 0.98 | 100 |  | fgi | 0.95 | 100 |  | fgi | 0.93 | 100 |  | fgi | 1.01 | 100 |
| bcd | 1 |  | | bcd | 0.92 | 100 |  | bcd | 0.97 | 100 |  | bcd | 0.96 | 100 |  | bcd | 0.95 | 100 |  | bcd | 0.99 | 100 |
| hjkl | 1 |  | | hjkl | 0.93 | 100 |  | hjkl | 0.99 | 100 |  | hjkl | 0.96 | 100 |  | hjkl | 0.93 | 100 |  | hjkl | 1.00 | 100 |
| bdce | 1 |  | | bdce | 0.91 | 100 |  | bdce | 0.97 | 100 |  | bdce | 0.93 | 100 |  | bdce | 0.92 | 100 |  | bdce | 0.96 | 100 |
| abcde | 2 |  | | abcde | 1.82 | 100 |  | abcde | 1.94 | 100 |  | abcde | 1.86 | 100 |  | abcde | 1.85 | 100 |  | abcde | 1.92 | 100 |
|  |  |  | | ae | 0.04 | 26 |  | ae | 0.04 | 26 |  | ai | 0.03 | 16 |  | kl | 0.02 | 11 |  |  |  |  |
|  |  |  | | al | 0.03 | 10 |  | al | 0.03 | 10 |  | cd | 0.01 | 30 |  | gi | 0.02 | 10 |  |  |  |  |
|  |  |  | |  |  |  |  |  |  |  |  | kl | 0.03 | 32 |  | bc | 0.01 | 15 |  |  |  |  |
|  |  |  | |  |  |  |  |  |  |  |  | gi | 0.03 | 29 |  | ae | 0.02 | 10 |  |  |  |  |
|  |  |  | |  |  |  |  |  |  |  |  | fi | 0.02 | 34 |  |  |  |  |  |  |  |  |
|  |  |  | |  |  |  |  |  |  |  |  | hk | 0.02 | 31 |  |  |  |  |  |  |  |  |
|  |  |  | |  |  |  |  |  |  |  |  | be | 0.02 | 12 |  |  |  |  |  |  |  |  |
|  |  |  | |  |  |  |  |  |  |  |  | jk | 0.02 | 36 |  |  |  |  |  |  |  |  |
|  |  |  | |  |  |  |  |  |  |  |  | bc | 0.02 | 40 |  |  |  |  |  |  |  |  |
|  |  |  | |  |  |  |  |  |  |  |  | al | 0.03 | 13 |  |  |  |  |  |  |  |  |
|  |  |  | |  |  |  |  |  |  |  |  | ce | 0.02 | 25 |  |  |  |  |  |  |  |  |
|  |  |  | |  |  |  |  |  |  |  |  | ae | 0.03 | 40 |  |  |  |  |  |  |  |  |
|  |  |  | |  |  |  |  |  |  |  |  | hkl | 0.02 | 16 |  |  |  |  |  |  |  |  |
|  |  |  | |  |  |  |  |  |  |  |  | bde | 0.03 | 32 |  |  |  |  |  |  |  |  |
|  |  |  | |  |  |  |  |  |  |  |  | hjl | 0.02 | 29 |  |  |  |  |  |  |  |  |
|  |  |  | |  |  |  |  |  |  |  |  | jkl | 0.02 | 10 |  |  |  |  |  |  |  |  |
|  |  |  | |  |  |  |  |  |  |  |  | bce | 0.01 | 16 |  |  |  |  |  |  |  |  |
|  |  |  | |  |  |  |  |  |  |  |  | ace | 0.01 | 11 |  |  |  |  |  |  |  |  |
|  |  |  | |  |  |  |  |  |  |  |  | cde | 0.02 | 13 |  |  |  |  |  |  |  |  |
|  |  |  | |  |  |  |  |  |  |  |  | afgi | 0.03 | 19 |  |  |  |  |  |  |  |  |
|  |  |  | |  |  |  |  |  |  |  |  | abde | 0.02 | 15 |  |  |  |  |  |  |  |  |
|  |  |  | |  |  |  |  |  |  |  |  | fgil | 0.02 | 23 |  |  |  |  |  |  |  |  |
|  |  |  | |  |  |  |  |  |  |  |  | efgi | 0.02 | 10 |  |  |  |  |  |  |  |  |
|  |  |  | |  |  |  |  |  |  |  |  | abcd | 0.04 | 24 |  |  |  |  |  |  |  |  |
|  |  |  | |  |  |  |  |  |  |  |  | fgik | 0.02 | 13 |  |  |  |  |  |  |  |  |
|  |  |  | |  |  |  |  |  |  |  |  | ahjk | 0.02 | 11 |  |  |  |  |  |  |  |  |
|  |  |  | |  |  |  |  |  |  |  |  | bcdel | 0.03 | 13 |  |  |  |  |  |  |  |  |
|  |  |  | |  |  |  |  |  |  |  |  | hijkl | 0.03 | 22 |  |  |  |  |  |  |  |  |
|  |  |  | |  |  |  |  |  |  |  |  | ahjkl | 0.02 | 23 |  |  |  |  |  |  |  |  |
|  |  |  | |  |  |  |  |  |  |  |  | ghjkl | 0.02 | 14 |  |  |  |  |  |  |  |  |
|  |  |  | |  |  |  |  |  |  |  |  | fgikl | 0.01 | 10 |  |  |  |  |  |  |  |  |
|  |  |  | |  |  |  |  |  |  |  |  | bcdei | 0.02 | 10 |  |  |  |  |  |  |  |  |
|  |  |  | |  |  |  |  |  |  |  |  | abcdei | 0.03 | 16 |  |  |  |  |  |  |  |  |
|  |  |  | |  |  |  |  |  |  |  |  | abcdel | 0.03 | 24 |  |  |  |  |  |  |  |  |
|  |  |  | |  |  |  |  |  |  |  |  | abcdef | 0.02 | 11 |  |  |  |  |  |  |  |  |

*The column “True” represents the real phylogenetic history of the tree data and the other columns represents the reconstructed results by five methods. Furthermore, “WV” denotes the average weight in all 100 runs; “BV” denotes the bootstrap value.

**Supplementary Table S2.** True splits and splits reconstructed from the phylogenetic network with three reticulate events.

| **True** | |  | **QS-Net** | | |  | **Quartet-Net** | | |  | **Neighbor-Net** | | |  | **Split-Decomposition** | | |  | **Neighbor-Joining** | | |
| --- | --- | --- | --- | --- | --- | --- | --- | --- | --- | --- | --- | --- | --- | --- | --- | --- | --- | --- | --- | --- | --- |
| **Split** | **WV** |  | **Split** | **WV** | **BV** |  | **Split** | **WV** | **BV** |  | **Split** | **WV** | **BV** |  | **Split** | **WV** | **BV** |  | **Split** | **WV** | **BV** |
| ab | 4 |  | ab | 4 | 100 |  | ab | 4 | 100 |  | ab | 4 | 100 |  | ab | 4 | 48 |  | ab | 4 | 100 |
| bc | 2 |  | bc | 2.05 | 100 |  | bc | 2.05 | 100 |  | bc | 5.05 | 100 |  | bc | 4.01 | 61 |  | bc |  |  |
| bd | 4 |  | bd | 3.99 | 100 |  | bd | 3.99 | 100 |  | bd |  |  |  | bd | 6.67 | 90 |  | bd |  |  |
| ce | 4 |  | ce | 4.04 | 100 |  | ce | 4.04 | 100 |  | ce | 7.80 | 100 |  | ce | 53.91 | 100 |  | ce | 5.20 | 100 |
| df | 4 |  | df | 3.93 | 100 |  | df | 3.93 | 100 |  | df | 8.15 | 89 |  | df | 56.13 | 100 |  | df |  |  |
| fg | 2 |  | fg | 2.00 | 100 |  | fg | 2.00 | 100 |  | fg | 7.05 | 100 |  | fg | 54.34 | 100 |  | fg | 4.71 | 100 |
| gh | 4 |  | gh | 3.92 | 100 |  | gh | 3.92 | 100 |  | gh | 8.13 | 9 |  | gh | 55.92 | 100 |  | gh |  |  |
| hj | 4 |  | hj | 3.91 | 100 |  | hj | 3.91 | 100 |  | hj | 7.25 | 100 |  | hj | 55.65 | 100 |  | hj |  |  |
| jk | 4 |  | jk | 3.90 | 100 |  | jk | 3.90 | 100 |  | jk | 7.16 | 100 |  | jk | 56.31 | 100 |  | jk | 6.33 | 100 |
| jkl | 4 |  | jkl | 3.51 | 100 |  | jkl | 3.73 | 100 |  | jkl | 6.84 | 100 |  | jkl | 52.8 | 100 |  | jkl | 6.87 | 100 |
| hjk | 4 |  | hjk | 3.60 | 100 |  | hjk | 3.67 | 100 |  | hjk | 7.05 | 100 |  | hjk | 54.2 | 100 |  | hjk |  |  |
| fgi | 2 |  | fgi | 1.75 | 100 |  | fgi | 1.87 | 100 |  | fgi | 0.10 | 3 |  | fgi | 1.82 | 24 |  | fgi | 1.74 | 100 |
| fgh | 2 |  | fgh | 1.83 | 100 |  | fgh | 1.83 | 100 |  | fgh | 6.06 | 9 |  | fgh | 3.42 | 57 |  | fgh |  |  |
| abd | 4 |  | abd | 3.58 | 100 |  | abd | 3.70 | 100 |  | abd | 8.04 | 100 |  | abd | 3.30 | 28 |  | abd |  |  |
| bcd | 2 |  | bcd | 1.81 | 100 |  | bcd | 1.84 | 100 |  | bcd |  |  |  | bcd | 2.22 | 30 |  | bcd |  |  |
| bce | 2 |  | bce | 1.82 | 100 |  | bce | 1.79 | 100 |  | bce | 6.00 | 100 |  | bce |  |  |  | bce |  |  |
| dfg | 2 |  | dfg | 1.81 | 100 |  | dfg | 1.83 | 100 |  | dfg | 5.84 | 91 |  | dfg | 3.24 | 61 |  | dfg |  |  |
| hjkl | 4 |  | hjkl | 3.44 | 100 |  | hjkl | 3.63 | 100 |  | hjkl | 6.92 | 100 |  | hjkl | 52.00 | 100 |  | hjkl | 5.52 | 100 |
| fghi | 2 |  | fghi | 1.74 | 100 |  | fghi | 1.81 | 100 |  | fghi | 6.58 | 96 |  | fghi | 2.71 | 29 |  | fghi |  |  |
| bcde | 2 |  | bcde | 1.77 | 100 |  | bcde | 1.75 | 100 |  | bcde |  |  |  | bcde |  |  |  | bcde |  |  |
| abce | 8 |  | abce | 6.95 | 100 |  | abce | 7.33 | 100 |  | abce | 9.34 | 100 |  | abce | 51.99 | 100 |  | abce | 9.11 | 100 |
| dfgh | 2 |  | dfgh | 1.77 | 100 |  | dfgh | 1.77 | 100 |  | dfgh |  |  |  | dfgh | 1.67 | 34 |  | dfgh |  |  |
| dfgi | 2 |  | dfgi | 1.70 | 100 |  | dfgi | 1.78 | 100 |  | dfgi | 1.49 | 57 |  | dfgi | 1.89 | 19 |  | dfgi |  |  |
| abcde | 8 |  | abcde | 6.89 | 100 |  | abcde | 7.33 | 100 |  | abcde | 17.58 | 100 |  | abcde | 131.81 | 100 |  | abcde | 11.32 | 100 |
| dfghi | 2 |  | dfghi | 1.67 | 100 |  | dfghi | 1.80 | 100 |  | dfghi | 7.22 | 100 |  | dfghi | 50.74 | 100 |  | dfghi |  |  |
|  |  |  | ae | 0.24 | 62 |  | ae | 0.24 | 62 |  | el | 0.14 | 19 |  | abcd | 1.21 | 16 |  |  |  |  |
|  |  |  | il | 0.2 | 31 |  | il | 0.2 | 31 |  | kl | 0.13 | 40 |  |  |  |  |  |  |  |  |
|  |  |  | be | 0.18 | 12 |  | be | 0.18 | 12 |  | cel | 0.12 | 26 |  |  |  |  |  |  |  |  |
|  |  |  | ac | 0.2 | 12 |  | ac | 0.2 | 12 |  | ekl | 0.06 | 10 |  |  |  |  |  |  |  |  |
|  |  |  |  |  |  |  |  |  |  |  | abcd | 2 | 100 |  |  |  |  |  |  |  |  |
|  |  |  |  |  |  |  |  |  |  |  | cekl | 0.1 | 16 |  |  |  |  |  |  |  |  |
|  |  |  |  |  |  |  |  |  |  |  | ejkl | 0.13 | 19 |  |  |  |  |  |  |  |  |
|  |  |  |  |  |  |  |  |  |  |  | abdf | 0.07 | 11 |  |  |  |  |  |  |  |  |
|  |  |  |  |  |  |  |  |  |  |  | cejkl | 0.12 | 21 |  |  |  |  |  |  |  |  |
|  |  |  |  |  |  |  |  |  |  |  | ehjkl | 0.13 | 27 |  |  |  |  |  |  |  |  |
|  |  |  |  |  |  |  |  |  |  |  | fghij | 0.14 | 22 |  |  |  |  |  |  |  |  |
|  |  |  |  |  |  |  |  |  |  |  | abcel | 0.13 | 14 |  |  |  |  |  |  |  |  |
|  |  |  |  |  |  |  |  |  |  |  | abdfgi | 0.13 | 28 |  |  |  |  |  |  |  |  |
|  |  |  |  |  |  |  |  |  |  |  | abcekl | 0.11 | 19 |  |  |  |  |  |  |  |  |
|  |  |  |  |  |  |  |  |  |  |  | abcdel | 0.19 | 13 |  |  |  |  |  |  |  |  |
|  |  |  |  |  |  |  |  |  |  |  | abcdef | 1.56 | 19 |  |  |  |  |  |  |  |  |

**Supplementary Table S3.** True splits and splits reconstructed from the phylogenetic network with five reticulate events.

| **True** | |  | **QS-Net** | | |  | **Quartet-Net** | | |  | **Neighbor-Net** | | |  | **Split-Decomposition** | | |  | **Neighbor-Joining** | | |
| --- | --- | --- | --- | --- | --- | --- | --- | --- | --- | --- | --- | --- | --- | --- | --- | --- | --- | --- | --- | --- | --- |
| **Split** | **WV** |  | **Split** | **WV** | **BV** |  | **Split** | **WV** | **BV** |  | **Split** | **WV** | **BV** |  | **Split** | **WV** | **BV** |  | **Split** | **WV** | **BV** |
| ab | 12 |  | ab | 11.9 | 100 |  | ab | 11.9 | 100 |  | ab | 4.59 | 94 |  | ab |  |  |  | ab | 6.37 | 52 |
| bc | 8 |  | bc | 8.11 | 100 |  | bc | 8.11 | 100 |  | bc | 11.18 | 100 |  | bc | 7.95 | 100 |  | bc |  |  |
| bd | 16 |  | bd | 15.94 | 100 |  | bd | 15.94 | 100 |  | bd | 28.02 | 11 |  | bd | 4.70 | 100 |  | bd |  |  |
| ce | 16 |  | ce | 16 | 100 |  | ce | 16 | 100 |  | ce | 16 | 100 |  | ce | 16 | 100 |  | ce | 16 | 100 |
| df | 16 |  | df | 15.67 | 100 |  | df | 15.67 | 100 |  | df | 15.65 | 100 |  | df | 16.83 | 100 |  | df |  |  |
| fg | 8 |  | fg | 9.88 | 100 |  | fg | 9.88 | 100 |  | fg | 11.3 | 100 |  | fg | 12.36 | 100 |  | fg | 13.51 | 100 |
| gh | 16 |  | gh | 15.73 | 100 |  | gh | 15.73 | 100 |  | gh | 15.6 | 100 |  | gh | 16.87 | 100 |  | gh |  |  |
| hj | 16 |  | hj | 15.95 | 100 |  | hj | 15.95 | 100 |  | hj | 27.86 | 5 |  | hj | 4.86 | 100 |  | hj |  |  |
| ij | 12 |  | ij | 11.82 | 100 |  | ij | 11.82 | 100 |  | ij | 13.00 | 89 |  | ij |  |  |  | ij | 6.38 | 44 |
| jk | 8 |  | jk | 8.10 | 100 |  | jk | 8.10 | 100 |  | jk | 12.64 | 100 |  | jk | 7.93 | 100 |  | jk |  |  |
| kl | 16 |  | kl | 15.97 | 100 |  | kl | 15.97 | 100 |  | kl | 18.78 | 100 |  | kl | 16.02 | 100 |  | kl | 16.15 | 100 |
| jkl | 8 |  | jkl | 7.18 | 100 |  | jkl | 7.12 | 100 |  | jkl | 10.36 | 100 |  | jkl | 0.31 | 32 |  | jkl | 3.90 | 56 |
| hjk | 8 |  | hjk | 7.28 | 100 |  | hjk | 7.41 | 100 |  | hjk | 3.69 | 14 |  | hjk |  |  |  | hjk |  |  |
| ihj | 12 |  | ihj | 10.87 | 100 |  | ihj | 11.08 | 100 |  | ihj | 11.92 | 46 |  | ihj |  |  |  | ihj |  |  |
| fgi | 2 |  | fgi | 1.64 | 100 |  | fgi |  |  |  | fgi |  |  |  | fgi |  |  |  | fgi |  |  |
| fgh | 8 |  | fgh | 7.36 | 100 |  | fgh | 6.02 | 100 |  | fgh | 11.01 | 100 |  | fgh | 8.28 | 100 |  | fgh |  |  |
| abd | 12 |  | abd | 10.79 | 100 |  | abd | 10.99 | 100 |  | abd | 11.83 | 93 |  | abd |  |  |  | abd |  |  |
| bcd | 8 |  | bcd | 7.34 | 100 |  | bcd | 7.49 | 100 |  | bcd | 3.65 | 6 |  | bcd |  |  |  | bcd |  |  |
| bce | 8 |  | bce | 7.14 | 100 |  | bce | 7.09 | 100 |  | bce | 14.71 | 100 |  | bce | 0.32 | 31 |  | bce | 3.97 | 48 |
| afg | 2 |  | afg | 1.65 | 100 |  | afg |  |  |  | afg |  |  |  | afg |  |  |  | afg |  |  |
| dfg | 8 |  | dfg | 7.34 | 100 |  | dfg | 6.01 | 100 |  | dfg | 9.73 | 100 |  | dfg | 8.33 | 100 |  | dfg |  |  |
| hjkl | 8 |  | hjkl | 7.18 | 100 |  | hjkl | 6.92 | 100 |  | hjkl | 7.94 | 44 |  | hjkl |  |  |  | hjkl |  |  |
| ijkl | 16 |  | ijkl | 13.75 | 100 |  | ijkl | 14.3 | 100 |  | ijkl | 5.02 | 59 |  | ijkl | 0.23 | 11 |  | ijkl | 11.89 | 100 |
| fghi | 2 |  | fghi | 1.36 | 100 |  | fghi | 1.43 | 100 |  | fghi |  |  |  | fghi |  |  |  | fghi |  |  |
| adfg | 2 |  | adfg | 1.37 | 100 |  | adfg | 1.48 | 100 |  | adfg |  |  |  | adfg |  |  |  | adfg |  |  |
| bdce | 8 |  | bdce | 7.08 | 100 |  | bdce | 6.89 | 100 |  | bdce | 8.51 | 1 |  | bdce |  |  |  | bdce |  |  |
| afgh | 2 |  | afgh | 1.60 | 100 |  | afgh | 1.47 | 100 |  | afgh |  |  |  | afgh |  |  |  | afgh |  |  |
| abce | 16 |  | abce | 13.76 | 100 |  | abce | 14.29 | 100 |  | abce | 5.80 | 99 |  | abce | 0.58 | 14 |  | abce | 11.48 | 100 |
| dfgh | 8 |  | dfgh | 7.07 | 100 |  | dfgh | 7.21 | 100 |  | dfgh | 9.23 | 100 |  | dfgh | 4.33 | 100 |  | dfgh |  |  |
| dfgi | 2 |  | dfgi | 1.64 | 100 |  | dfgi | 1.53 | 100 |  | dfgi |  |  |  | dfgi |  |  |  | dfgi |  |  |
| ihjkl | 16 |  | ihjkl | 13.76 | 100 |  | ihjkl | 14.4 | 100 |  | ihjkl | 24.15 | 100 |  | ihjkl | 23.59 | 100 |  | ihjkl | 17.00 | 100 |
| abdfg | 4 |  | abdfg | 3.20 | 100 |  | abdfg | 3.30 | 100 |  | abdfg | 5.88 | 93 |  | abdfg | 0.60 | 46 |  | abdfg |  |  |
| abcde | 16 |  | abcde | 13.72 | 100 |  | abcde | 14.37 | 100 |  | abcde | 24.82 | 100 |  | abcde | 23.28 | 100 |  | abcde | 16.62 | 100 |
| fghij | 4 |  | fghij | 3.17 | 100 |  | fghij | 3.25 | 100 |  | fghij | 5.67 | 45 |  | fghij | 0.55 | 43 |  | fghij |  |  |
| adfgh | 2 |  | adfgh | 1.43 | 100 |  | adfgh | 1.53 | 100 |  | adfgh |  |  |  | adfgh |  |  |  | adfgh |  |  |
| dfghi | 2 |  | dfghi | 1.39 | 100 |  | dfghi | 1.49 | 100 |  | dfghi |  |  |  | dfghi |  |  |  | dfghi |  |  |
| abdfgh | 4 |  | abdfgh | 3.22 | 100 |  | abdfgh | 3.20 | 100 |  | abdfgh | 5.05 | 93 |  | abdfgh | 3.26 | 100 |  | abdfgh |  |  |
| abcekl | 4 |  | abcekl | 3.23 | 100 |  | abcekl | 3.22 | 100 |  | abcekl | 5.40 | 46 |  | abcekl | 3.22 | 100 |  | abcekl |  |  |
|  |  |  | ae | 0.85 | 93 |  | ae | 0.85 | 93 |  | abcd | 4.16 | 93 |  | abcd | 0.12 | 12 |  |  |  |  |
|  |  |  | il | 0.86 | 85 |  | il | 0.86 | 85 |  | hijk | 3.83 | 43 |  |  |  |  |  |  |  |  |
|  |  |  | ik | 0.70 | 14 |  | ik | 0.70 | 14 |  | abcdfg | 0.21 | 33 |  |  |  |  |  |  |  |  |
|  |  |  | al | 0.65 | 12 |  | al | 0.65 | 12 |  | abcdef | 0.27 | 35 |  |  |  |  |  |  |  |  |
